# Epidemiology of carpal fractures: is it only about the scaphoid?

**DOI:** 10.1007/s00068-022-02213-5

**Published:** 2023-01-17

**Authors:** Olivia Boeddrich, Anna Lena Sander, Thomas Lustenberger, Ingo Marzi, Johannes Frank, Maika Voth, Katharina Sommer

**Affiliations:** 1 Department of Trauma, Hand and Reconstructive Surgery, Hospital of the Johann Wolfgang Goethe-University, Theodor Stern Kai 7, 60590 Frankfurt Am Main, Germany; 2 Department of Trauma Surgery, Kantonsspital Aarau, Tellstraße 25, 5001 Aarau, Switzerland

**Keywords:** Carpal fracture, Epidemiology, Conservative treatment, Operative treatment

## Abstract

Because of their low incidence, studies about carpal fractures are rare. The aim of the present study was to analyze epidemiology and treatment of fractured carpal bones. We retrospectively analyzed data of 178 patients admitted to our emergency room with carpal fractures over 6 years. More males than woman were injured. In 91%, a CT scan was performed. The most commonly affected bone was the triquetrum followed by the scaphoid. Almost all triquetral fractures were treated conservatively as opposed to perilunate dislocations that were all operated on. Half of all patients with scaphoid fractures were operated. Young men had the highest risk to sustain a carpal fracture. The triquetrum and the scaphoid are most frequently affected. Usually a CT scan is needed. Treatment of scaphoid and perilunate luxation fractures is rather operative whereas the other fractures mostly allow conservative casting. Nevertheless, correct indication for treatment is important to avoid sequelae.

## Introduction

Hand fractures comprise about a fifth of all fractures treated in an emergency setting, resulting in an annual incidence of about 3–4/100,000. Within these, 8% are fractures of the carpal bones. [1]. The fractures of the carpal bones are rare compared to the fractures of the metacarpals and the phalanxes [2]. They comprise about 8% of these fractures [3].

Most studies focus on diagnostic and treatment options for scaphoid fractures as these are stated to be the most common among the carpal bones [4–10]. Just a few publications address the incidence and treatment options for the fractures of the other carpals [11–13]. Due to their outstanding importance for overall hand function, missed carpal fractures may cause pain, dysfunction, and loss of productivity making carpal fractures an important issue [2].

The anatomy of the carpus is complex consisting of two rows of carpal bones. The proximal row includes the scaphoid, the lunate, the triquetrum, the latter with the adjacent pisiforme as a volar sesamoid bone. The second row contains the trapezium, trapezoid, capitate, and the hamate. Because of this complex anatomy, usually a CT scan is needed for correct diagnosis for carpal fractures as they might be missed on conventional radiographic pictures [13].

This study focuses on the overall incidence of fractures of the different carpal bones and their treatment strategies based on current epidemiological data.

## Patients and methods

After attaining institutional review board approval (GN239/16), we retrospectively analyzed the occurrence of the different carpal fractures and the method of treatment used in patients admitted to our emergency room in the period between 2013 and 2018, retrospectively. All patients included were ≥ 18 years old. We performed a computer based ICD-10 search as well as a search in our radiological reports for fractures of each carpal bone. Overall, carpal fractures occurred in 178 of the 16,157 patients who were admitted to our emergency room during this period.

Data were obtained by analysis of the institution’s data-base, charts, and radiological examinations. This included age, sex, injury etiology, fracture type, and treatment methods.

Trauma mechanism as low-energy trauma (i.e., fall from standing or seating height, crushing injury, hyperextension, distortions) and high-energy trauma (i.e., motor vehicle accident, bicycle accident, fall from high altitude) were noted.

## Results

### Patient and injury details

**Fig. 1:**
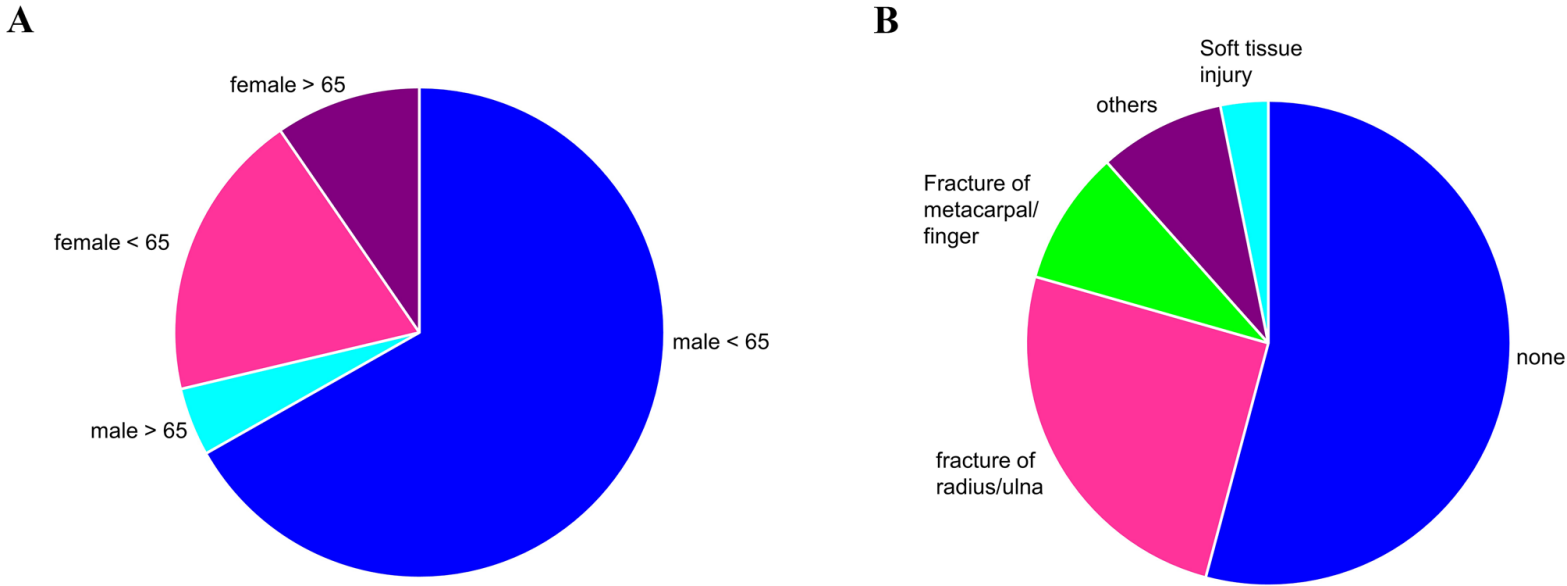
**A** Age distribution**:** 66.8% of patients suffering from carpal fracture are male below the age of 65 years; 19,1% of patients were female below 65 years; less frequent are carpal fractures in mal > 65 with 4.5% and female > 65 with 9.6%. **B** Concomitant injuries: in most carpal fractures no concomitant injury was observed (57.9%), most frequent occurring concomitant injury was fracture of the radius or ulna (27.0%) followed by ligament ruptures (9.6%), fractures of metacarpals or fingers (9%), soft tissue injuries were rare (1.1%)

In the period of 2013 to 2018, 178 patients (1.1% of all patients admitted to our emergency room) were diagnosed with a carpal fracture having in sum 229 carpal fractures. Of these 28.7% (51) were female and 71.3% (127) male. The mean age was 43.6 years ranging from 18 to 89 years. 86% of the patients were younger than 65 years and subsequently 14% older than 65 years. In the male population, almost all patients (93.7%) were younger than 65. Only 6.3% were older than 65. In female population, two-third (66.7%) were younger than 65 and 33.3% older (Fig. 1A).

Distribution between sides was almost equal with 50% (89) injuries on the right hand and 47.8% (85) on the left hand. Carpal fractures of both hands were only seen in 2.2% (4). Most of the patients were diagnosed with just one carpal fracture (143; 80.3%). Only 35 (19.7%) patients had multiple carpal fractures.

More than half of all patients (57.5%) had no accompanying injuries (Fig. 1B). The remaining 42.5% of patients suffered an additional hand related injury. Most common were fractures of the distal radius and ulna with 27%, ligamentous injuries with 9.6% and metacarpal or phalangeal fractures in 9% (Fig. 1B). Accompanying soft tissue injuries were rare with only 1.1%. Other injuries were diagnosed in 3.4%. 15.7% of all fractures were due to industrial accidents.

**Fig. 2:**
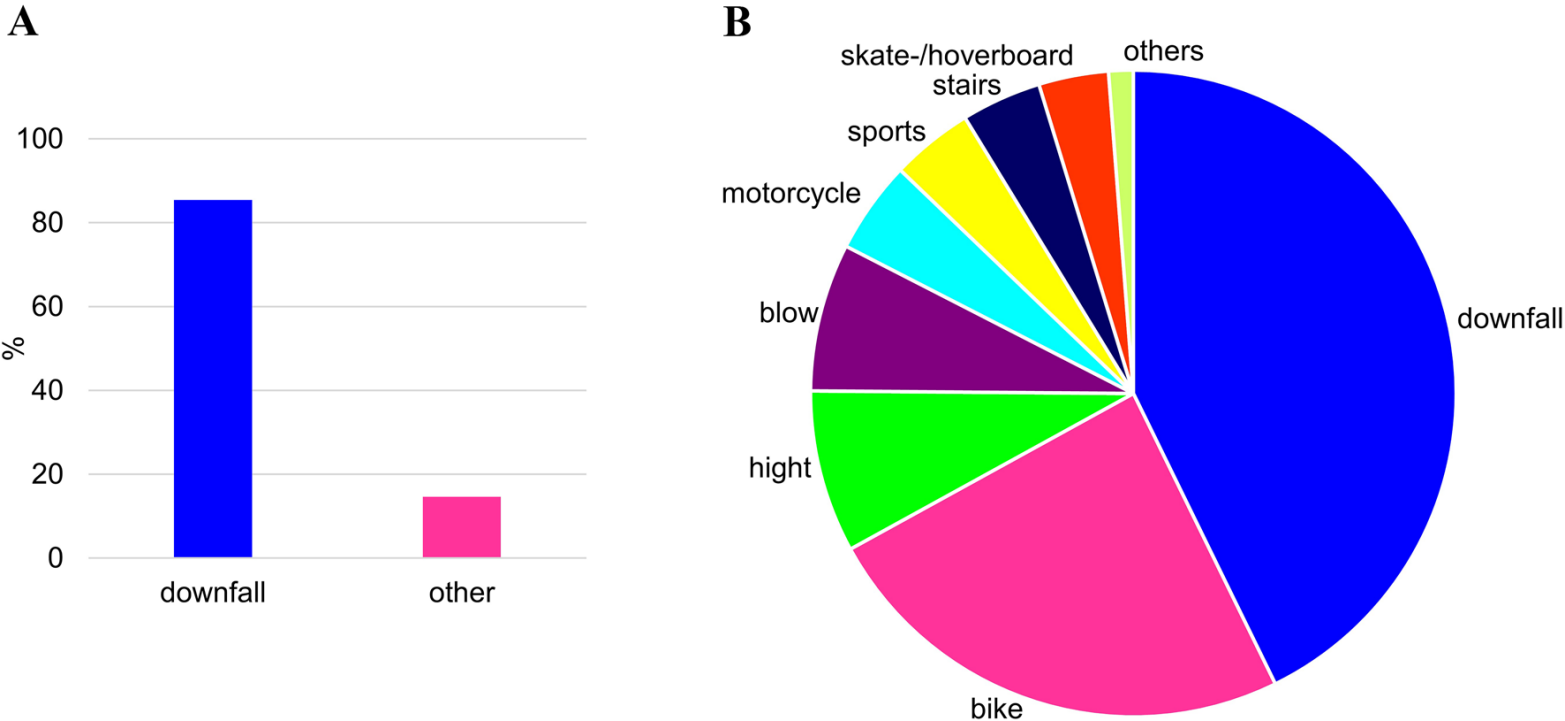
Trauma mechanism for carpal fractures: 85.4% of carpal fractures are caused by a downfall on the hand of any kind (**A**); more specifically the three most often causing trauma mechanisms are downfall from standing (41.6%) followed by fall from a bike (23.6%) and fall from high altitude (7.9%), less frequent were motorcycle accidents (4.5%), falling from stairs (3.9%) or falling from skate- or Hoverboard (3.4%)

Most of the injuries were due to a downfall on the hand (85.4%) (Fig. 2B). Only 14.6% were based on a different trauma mechanism like circular saw injury or violent dispute. A normal downfall on the hand (41.6%) was the most common cause for carpal fractures followed by followed by falls from the bike (23.6%), from high altitude (7.9%) from the motorcycle (4.5%), from the stairs (3.9%) or from the skate- or hoverboard (3.4%) summing up to a total of 43.3% of patients sustaining a high energy trauma (Fig. 2B).

For diagnosis, the majority of the patients received a CT scan (91%). Only conventional X-ray pictures were taken in the remaining patients with 9%. MRI scan was additionally performed in 5.6%. Usually, the additional MRI as performed when concomitant ligament injuries were suspected. Only in one case the MRI was used to control a scaphoid fracture.

### Fracture distribution

**Fig. 3:**
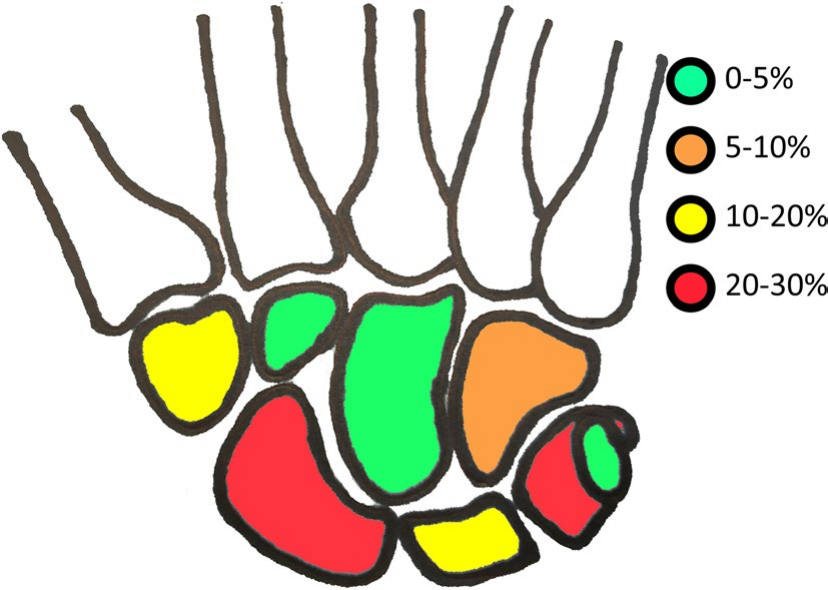
Heat map for distribution of carpal bone fractures: most commonly involved are the triquetrum and scaphoid with 2.8% and 27.9% respectively followed by hamate fractures with 11.4%; less frequently involved are trapezium (9.6%), lunate (6.6%), capitate (4.4%), pisiform (4.4%), and trapezoid (3.1%); perilunate luxations occur in 3.9%

The most common diagnosed fracture was of the os triquetrum with 28.8% followed by the scaphoid fracture with 27.9% both together add up to more than half of all fractures (Fig. 3). Also frequent were fractures of the hamate and the trapezium with 11.4% and 9.6% respectively (Fig. 3). Fractures of the pisiform, the capitate, the trapezoid, and the lunate as well as perilunate fracture dislocation were rare with 6.6% for lunate, 4.4% for pisiform and capitate, 3.1% for trapezoid, and 3.9% for perilunate fracture dislocation (Fig. 3).

### Therapy

**Fig. 4:**
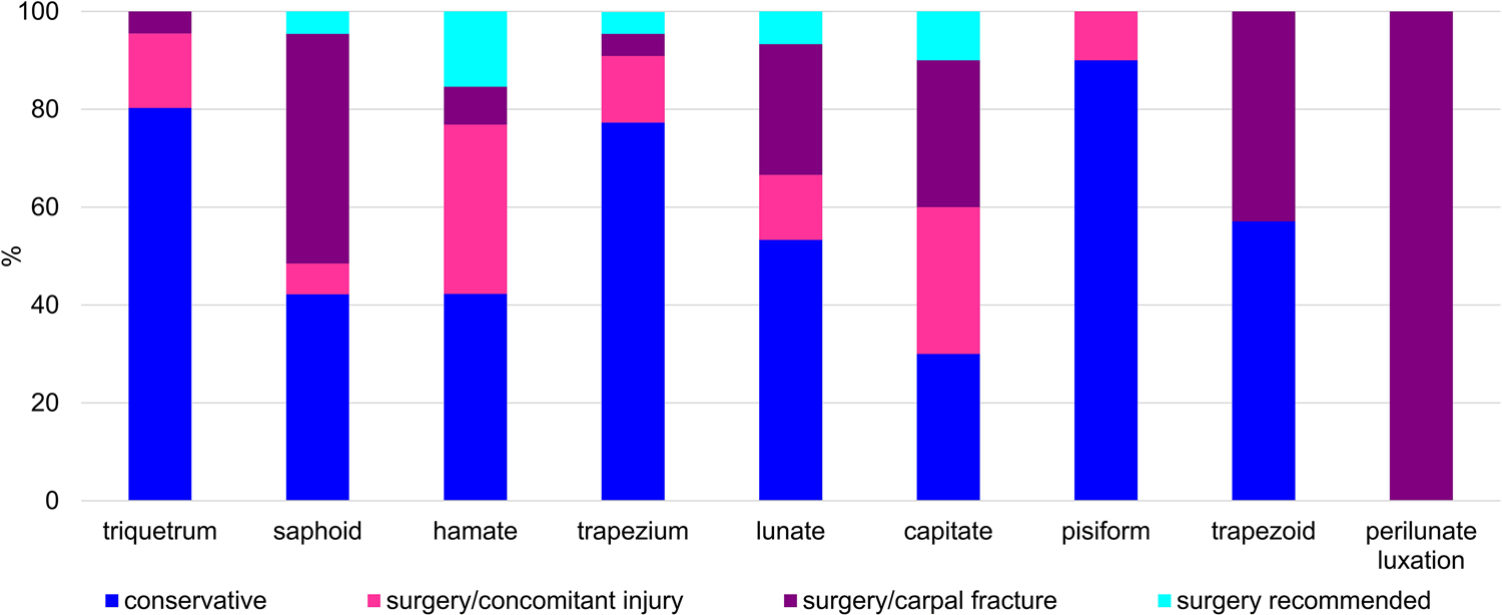
Choice of treatment for each carpal bone: fractures were either treated conservatively, received surgery due to concomitant fracture, underwent surgery because of the carpal fracture, or surgery was recommended, but not performed in our clinic

Most of the carpal fractures were treated conservatively with splint immobilization (62.5%). 19.1% of the patients underwent surgery because of the carpal fracture and 13.5% because of concomitant injury. In 5%, surgery was recommended but patients did not come back on the appointed date (Fig. 4).

All perilunate fracture dislocations were treated operatively. Of scaphoid fractures, more than half underwent surgery (53.2%). Of those only 6.3% had accompanying fractures. In 4.7% of the cases surgery was recommended but treatment was continued elsewhere. In capitate fractures, 60% of the patients underwent surgery. Half of them (30%) had also concomitant injuries that were treated operatively. Choice of treatment in lunatum fractures was almost equally distributed between operative and conservative treatment as 42.9% received surgery and 57.1% not. Of these 42.9% all surgeries were due to concomitant injuries (Fig. 4).

In contrast, fractures of the os triquetrum and the os trapezium were treated conservatively with 80.3% and 77.3%, respectively and the surgeries performed were most likely due to concomitant injury on the wrist or hand (15.2% and 13.6%). Trapezoid fractures underwent surgical intervention more often with 42.9%, but all interventions were due to concomitant injury. Fractures of the hamate were treated conservatively in 42.3% and surgery was primarily performed due to accompanying fractures (34.6%). Only 7.7% underwent surgery because of the hamate fracture itself. In pisiform fractures, only 10% (1) were treated operatively because of concomitant injury, the remaining fractures were treated conservatively (Fig. 4).

## Discussion

Carpal fractures are rare ranging below 8% of all hand fractures [3]. Disproportionally often they are found in young men. These representing two-thirds of the study population that suffer from these fractures (66.8%). Our results are similar to other epidemiological studies concerning the occurrence of carpal fractures. In earlier studies, the surplus of carpal fractures in the population of young male persons was mostly explained by their leisure time activities and sports [14]. Although young men are affected most frequently, these injuries are rarely related to industrial accidents (15.7%). This is supported by the fact, that a common cause for the injury mechanism is falling from bike, motorcycle, skate- or hoverboard that are usually leisure time activities.

In contrast to the literature, we did not find another peak of carpal fractures in older women, because women over the age of 65 just accounted for 9.6% of the fractures.

Otherwise, our findings were similar to literature as we noted concomitant injuries, like fracture of the distal radius or ulna, metacarpal and finger fractures in more than half of the cases. We also found that usually there is just one carpal fracture and just one hand involved. As most of the carpal fractures are caused by some kind of downfall, there is no preference to the side injured.

In our population almost all fractures were diagnosed by CT scan (91%). This is fairly explainable by the fact that on conventional radiography carpal fractures might not be seen [13]. For scaphoid fractures the sensitivity has been described as 70% and is surely below 50% for the other carpal fractures [2]. Thus in case of indistinct radiological finding or clinical suspicion a CT scan is performed in our clinic.

Usually, MRI is not needed for the diagnosis. In our study, only in rare cases a MRI was needed additionally. According to literature it was mostly performed if a concomitant soft tissue injury was suspected as TFCC lesions or scapholunate ligament ruptures. [15] Furthermore, MRI can be useful for occult scaphoid fractures, imaging in children or evaluation of the age of the fracture. [16, 17]. With regard to our study, MRI imaging is seldom needed in case of a carpal fracture.

Distribution of carpal fractures between the different bones vary greatly among the studies. Studies of Oh et al. (2015), van Onselen et al. (2003), and our own distribution of fractures of the different carpal bones are enlisted in Table 1 [3, 13]. Different study design might explain this high variety in fracture incidence of the different carpal bones. Whereas Oh et al. analyzed the 3D CT scans of the wrist, von Onselen analyzed the combined data from CT scans and clinical records. In our study we conducted a search of clinical records and clinically indicated CT scan and reports and verified the carpal fractures by a different observant. In our own data, we found high numbers of additionally verified carpal fractures in the radiological reports that were not noted in the clinical records. This fact leading to the assumption, that carpal fractures that do not need additional treatment might often be omitted in clinical records.

**Table 1:** Distribution of carpal bone fractures

Study	Triquetrum (%)	Scaphoid (%)	Hamate (%)	Capitate (%)	Lunate (%)	Pisiform (%)	Trapezoid (%)	Trapezium (%)
Oh	28	25	18	11	9	5	2	2
Van Onslen	28.6	61.4	4.3	1.4	1.4	0	1.4	1.4
Boeddrich	28.8	27.9	11.4	4.4	6.6	4.4	3.1	9.6

Comparison of the study of Oh et al. (2015), van Onselen et al. (2003), and our own study

This is supported by the fact that most of the carpal fractures do not need surgical intervention and simple immobilization is sufficient in most singular carpal fracture cases. In our study, only 19.1% of patients received operative treatment because of the carpal fracture and 62.5% were treated conservatively. Another 13.5% just underwent surgical procedures because of concomitant injuries that needed to be operated anyway. Just all perilunate fracture dislocations were treated operatively and there as a high number of operative treated scaphoid fractures as well (53.2%).

## Conclusion

Compared to the fractures of the metacarpals and phalanxes, carpal bones are less affected by fractures and occur most often in young men. Although literature is mainly focused on the scaphoid, in our study, the incidence of scaphoid fractures is second to triquetrum fractures.

Usually a CT scan is needed for correct diagnosis. Furthermore, proper treatment typically needs fracture analysis by CT scan. Thus, in cases of clinical suspicions a CT scan should be performed for a correct diagnosis to enable an adequate treatment to avoid compromising sequelae of the wrist.

## Data Availability

The data sets used and analyzed in the current study are available from the corresponding author on reasonable request.
